# Omega-3-Enriched Diet Improves Metabolic Profile in Prdx6-Deficient Mice Exposed to Microgravity

**DOI:** 10.3390/life13122245

**Published:** 2023-11-22

**Authors:** Francesca Pacifici, Aikaterini Andreadi, Roberto Arriga, Donatella Pastore, Barbara Capuani, Roberto Bonanni, David Della-Morte, Alfonso Bellia, Davide Lauro, Giulia Donadel

**Affiliations:** 1 Department of Systems Medicine, University of Rome Tor Vergata, 00133 Rome, Italy; francesca.pacifici@uniroma2.it (F.P.); andreadi@med.uniroma2.it (A.A.); arrigaroby@libero.it (R.A.); barbaracapuani73@gmail.com (B.C.); david.dellamorte@uniroma2.it (D.D.-M.); bellia@med.uniroma2.it (A.B.); d.lauro@med.uniroma2.it (D.L.); 2 Department of Medical Sciences, Fondazione Policlinico Tor Vergata, 00133 Rome, Italy; 3 Department of Human Sciences and Quality of Life Promotion, San Raffaele University, 00166 Rome, Italy; donatella.pastore@uniroma5.it; 4 Department of Biomedicine and Prevention, University of Rome Tor Vergata, 00133 Rome, Italy; roberto.bonanni1288@gmail.com; 5 Department of Neurology, Evelyn F. McKnight Brain Institute, Miller School of Medicine, University of Miami, Miami, FL 33136, USA; 6 Interdisciplinary Center for Advanced Studies on Lab-on-Chip and Organ-on-Chip Applications (ICLOC), University of Rome Tor Vergata, 00133 Rome, Italy; 7 Department of Clinical Sciences and Translational Medicine, University of Rome Tor Vergata, 00133 Rome, Italy

**Keywords:** glucose metabolism, space flight, aging model

## Abstract

Background: Space travel has always been one of mankind’s greatest dreams. Thanks to technological innovation, this dream is becoming more of a reality. Soon, humans (not only astronauts) will travel, live, and work in space. However, a microgravity environment can induce several pathological alterations that should be, at least in part, controlled and alleviated. Among those, glucose homeostasis impairment and insulin resistance occur, which can lead to reduced muscle mass and liver dysfunctions. Thus, it is relevant to shed light on the mechanism underlaying these pathological conditions, also considering a nutritional approach that can mitigate these effects. Methods: To achieve this goal, we used *Prdx6*^−/−^ mice exposed to Hindlimb Unloading (HU), a well-established experimental protocol to simulate microgravity, fed with a chow diet or an omega-3-enriched diet. Results: Our results innovatively demonstrated that HU-induced metabolic alterations, mainly related to glucose metabolism, may be mitigated by the administration of omega-3-enriched diet. Specifically, a significant improvement in insulin resistance has been reported. Conclusions: Although preliminary, our results highlight the importance of specific nutritional approaches that can alleviate microgravity-induced harmful effects. These findings should be considered soon by those planning trips around the earth.

## 1. Introduction

Since 1961, humans have been exploring the unknown beyond the Earth, while also studying its interaction with the Solar System and the Moon to improve Earth’s well-being [1]. In the present day, the commercial space industry, driven by human curiosity, has given rise to civilian spaceflight opportunities aimed to observe the Earth and the space around it. Accordingly, the National Aeronautics and Space Administration (NASA), has secured funding to develop space stations that will now host private travelers, and hold the potential to accommodate residents for both living and working purposes in the future [2]. However, it should be considered that exposure to microgravity predisposes individuals to several stressors that can have adverse effects on human health, resulting in pathological changes, like those observed during aging [3]. Specifically, microgravity induces alterations in liver carbohydrate metabolism, including insulin resistance, which can lead to a diabetogenic phenotype [4]. Insulin resistance contributes to increased gluconeogenesis, leading to hyperglycemia, through the up-regulation of the key enzyme phosphoenolpyruvate carboxykinase (PEPCK), in mice subjected to unloaded hind limbs for two months [5]. PEPCK is the first regulatory enzyme of gluconeogenesis that is inhibited by the activation of insulin signaling pathway [6]. Specifically, after the interaction between insulin and its receptor, a downstream cascade is initiated, resulting in the activation of Akt/PKB, a kinase that suppresses the expression of PEPCK [7]. It has been reported that the administration of omega-3 polyunsaturated fatty acids (PUFA), improves glucose metabolism by alleviating insulin resistance, and decreasing gluconeogenesis in a mouse model of metabolic syndrome induced by high-fat diet [8]. Furthermore, the consumption of omega-3 fatty acids was found to mitigate bone loss induced by space flight and bed rest [9]. However, studies conducted in animal models of both aging and metabolic alterations, in relation to potential civilian travelers, are currently lacking. The aim of our manuscript is to investigate the impact of an omega-3-enriched diet on mice with both aging and metabolic alterations when exposed to HU. To achieve this goal, we used Peroxiredoxin 6 knockout mice (*Prdx6*^−/−^) subjected to hindlimb unloading, a well-defined and widely accepted experimental procedure to simulate microgravity [10]. As previously demonstrated, *Prdx6*^−/−^ mice develop an early stage of diabetes, characterized by insulin resistance [11], and showed a premature phenotype of senescence [12], making them an intriguing choice for simulating the health status of some future civilian travelers; until now, civilian space travelers have displayed a mean age of 50 years old and may present or are likely to manifest some metabolic disorders that should be considered [13]. Our preliminary data showed that *Prdx6*^−/−^ mice subjected to microgravity exhibited impaired glucose homeostasis, which was partially reversed by an omega-3-enriched diet before exposure to microgravity. Although preliminary, these results highlight the importance of omega-3 supplementation for future civilian travelers to mitigate glucose-related alterations induced by microgravity during space flights.

## 2. Materials and Methods

### 2.1. Animal Models

For the present study, 5 eight-month-old male C57BL/6J wild-type (WT) mice and 5 *Prdx6*^−/−^ mice of mixed background (C57/BL6: 129) were obtained from The Jackson Laboratory (Bar Harbor, ME, USA), and maintained in a temperature-controlled room on a 12 h: 12 h light–dark cycle, with access to food and water ad libitum. The project has been reviewed and approved by the Animal-Welfare Body of the University of Rome “Tor Vergata”; according to the previous law in force at the time (D.Lgs. 116/1992, EU 609/86), the project was notified to the Ministry of Health that approved it, applying the rule of tacit consent.

### 2.2. Normal and Omega-3 Diet

For the experimental procedures, mice were divided into two groups, and fed with different diets for three months: 5 WT and 5 *Prdx6*^−/−^ mice were fed with a standard diet by Mucedola Srl (Settimo Milanese, Italy, code 4RF18, protein 16%, fat 2.5%, carbohydrates 55.5%, fiber 7.5%, ashes 6.5% and water 12%) and 5 WT and 5 *Prdx6*^−/−^ mice were fed with a diet enriched in omega-3 fatty acids with an n–6 to n–3 ratio of 1–5 by Mucedola Srl (Settimo Milanese, Italy code 4RF25MOD, protein 21.5%, fat 10.5% (2.5% omega-3, 0.5% omega-6), carbohydrates 60%, fiber 4%, ashes 7% and water 10%). Body weight was assessed before (WT: 36 ± 2; *Prdx6*^−/−^ 27.5 ± 6.5) and after omega-3-enriched diet (WT: 28 ± 2.9; *Prdx6*^−/−^ 25 ± 2.2). At the end of the experimental procedures, the animals were sacrificed by cervical dislocation.

### 2.3. Antiorthostatic Suspension

**Figure 1:**
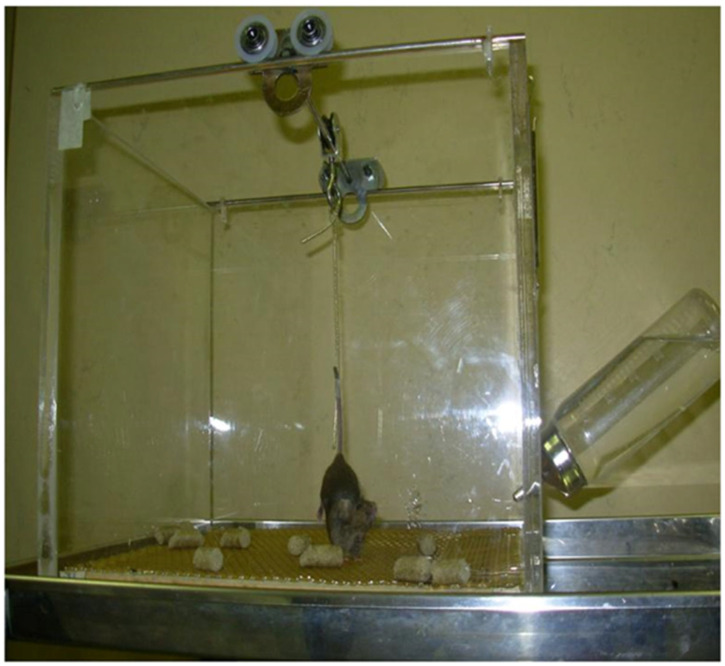
Hindlimb unloading to simulate microgravity.

Animal protocols using hindlimb unloading (HU) for 7 days have been approved by the NASAAmes Animal Care and Use Committee (ACUC) [10]. Briefly, mouse tail was cleaned with 70% ethanol and air-dried to remove any dead-skin residue. A strip of adhesive surgical tape (1.25 cm × 7 cm) was applied on the tail and the animal was suspended onto a swivel–pulley system that glides along a stainless steel rod that runs the length of the cage so that, even if the hind feet do not touch the cage floor, the animal was able to move freely throughout the area of the cage having free access to food and water. The angle of suspension was about 30° because it contributes to a uniform distribution of weight. Below the grid, used as a floor, is placed a paper towel to collect feces and urine (Figure 1).

### 2.4. Glucose Tolerance Tests and Measurement of Insulin Levels

The Intraperitoneal Glucose Tolerance Test (IPGTT) was performed as reported by Arriga et al. [14]. Briefly, all mice were fasted for 16 h and, then, injected with 2 g/kg body weight of glucose into the peritoneal cavity. Blood samples were collected from the retroorbital capillary plexus at 0, 15, 30, 60, 90, and 120 min (min). Blood glucose concentration was determined by using an automated OneTouch Lifescan Glucometer (Milpitas, CA, USA).

Insulin level was measured using a commercial Mouse Insulin ELISA kit (Mercodia, Uppsala, Sweden), following the manufacturer’s instructions. Briefly, sera obtained from IPGTT were loaded on a 96-well plate pre-coated with mouse monoclonal anti-insulin and then a Peroxidase-conjugated mouse monoclonal anti-insulin was added. The plate was incubated, with gentle shaking at room temperature for 2 h. Then, all the unbounded proteins and enzymes were washed out and the substrate TMB was added for 15 min. The reaction was stopped by adding 0.5 M H_2_SO_4_ and the optical density was analyzed by using the Multiskan FC microplate reader (ThermoFisher Scientific, Segrate, MI, Italy), at 450 nm.

### 2.5. Western Blot Assay

Proteins from the liver were extracted following Rea et al. [15]. Briefly, tissues were homogenized with a cold extraction buffer containing 20 mM Tris (pH 7.6), 137 mM NaCl, 1.5% Nonidet P40, 1 mM MgCl_2_, 1 mM CaCl_2_, 10% glycerol, phosphatases (Sigma Aldrich, St. Louis, MO, USA), and protease inhibitor cocktail (Roche, Indianapolis, IN, USA), and maintained in ice for 30 min. Then, they were centrifuged and supernatants were collected. Protein concentration was determined by Bradford assay (Bio-Rad Laboratories, Segrate, MI, Italy). Then, 50 µg of total protein was loaded in pre-cast 4–12% gels (ThermoFisher Scientific, Segrate, MI, Italy), separated by SDS PAGE, and transferred on a nitrocellulose membrane by using the Trans Turbo Blot Transfer System (Bio-Rad Laboratories, Segrate, MI, Italy). Membranes were blocked by using 5% Blotting Grade Blocker Non-Fat Dry Milk (Bio-Rad Laboratories, Segrate, MI, Italy), and incubated overnight at +4 °C with the following primary antibodies: anti-phospho Ser 473Akt-1, total Akt-1 (1:1000) (Cell Signaling Technology Inc., Danvers, MA, USA), anti-phosphoenolpyruvate carboxykinase (PEPCK) (1:1000) (Cayman Chemical, Ann Arbor, MI, Italy). The secondary antibodies were purchased from the Jackson ImmunoResearch and used at 1:10,000 (West Grove, PA, USA). The antigen–antibody complex was detected using ECL reagents (GE Healthcare, Chicago, IL, USA) and GelDoc Touch (Bio-Rad Laboratories, Segrate, MI, Italy). The immunoreactive bands were analyzed using ImageLab Software 6.1 (Bio-Rad Laboratories, Segrate, MI, Italy).

### 2.6. Gene Expression Analysis

Total RNA was isolated from frozen liver using Trizol reagents (Invitrogen Corp, Eugene, OR, USA) as reported by Pacifici et al., [12]. A total of 2.5 µg total RNA was reverse-transcribed into complementary DNA (cDNA) using the High Capacity cDNA Archive kit (Applied Biosystems, Foster City, CA, USA). Qualitative real-time polymerase chain reaction was performed using an ABI PRISM 7700 System, TaqMan reagents and inventoried patented primers: Mm01247058_m1 (PEPCK) (Applied Biosystems, Monza, Italy). The relative expression was calculated using the comparative ΔΔCT method, and the values were expressed as 2^−ΔΔCT^ [16].

### 2.7. Statistical Analysis

All data were analyzed using GraphPad Prism 9 (La Jolla, CA, USA). The results were reported as mean ± SD. Statistical analyses were performed using 2-way ANOVA. Values of *p* < 0.05 were considered statistically significant.

## 3. Results

### 3.1. Omega-3-Enriched Diet Improves Glucose Tolerance and Insulin Secretion in Microgravity Conditions

**Figure 2:**
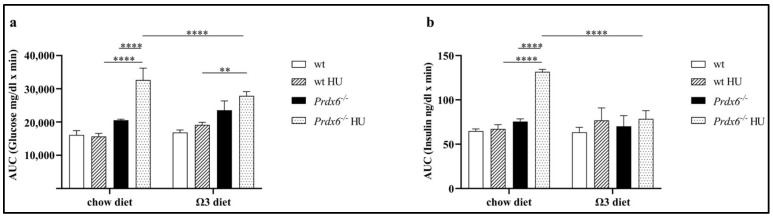
Glucose and Insulin levels following Intraperitoneal Glucose Tolerance Test IPGTT). (**a**) Area under the curve (AUC) related to glucose levels in wild type (wt) and *Prdx6*^−/−^ mice subjected or not to Hindlimb Unloaded (HU) and fed with chow or omega-3-enriched diet. (**b**) AUC related to insulin levels, in the same experimental conditions of (**a**). Data are expressed as mean ± SD from (*n* = 5). ** *p* < 0.005, **** *p* < 0.0001.

It has been reported that omega-3 administration improves glucose metabolism and lowers insulin levels in mice fed with a high-fat diet [17]. We have, now, evaluated the effect of an omega-3-enriched diet on glucose metabolism in our mouse model subjected to HU. As shown in Figure 2a, *Prdx6*^−/−^ mice subjected to HU, and fed with chow diet, displayed a significant increase in the glucose area under the curve (AUC) compared to both wt HU control mice and *Prdx6*^−/−^ mice (*p* < 0.0001), suggesting an impairment in glucose metabolism. However, when mice were subjected to an omega-3-enriched diet before HU, a decrease in glucose AUC was observed for *Prdx6*^−/−^ HU mice compared to both wt HU and *Prdx6*^−/−^ mice (*p* < 0.005 and *p* < 0.0001, respectively). Furthermore, we also analyzed the insulin AUC under the same experimental conditions. Similarly, to what has been observed for glucose AUC, also insulin AUC in *Prdx6*^−/−^ mice subjected to HU was significantly higher than in wt HU and *Prdx6*^−/−^ mice (*p* < 0.0001), suggesting a condition of insulin resistance (Figure 2b). However, an omega-3-enriched diet significantly reduced the insulin AUC in *Prdx6*^−/−^ HU mice compared to *Prdx6*^−/−^ HU fed a chow diet (*p* < 0.0001). These data suggest that an omega-3-enriched diet improves glucose metabolism when an alteration in glucose homeostasis exists.

### 3.2. Omega-3-Enriched Diet Promotes Insulin Signaling and Blunts Gluconeogenic Enzyme Expression

**Figure 3:**
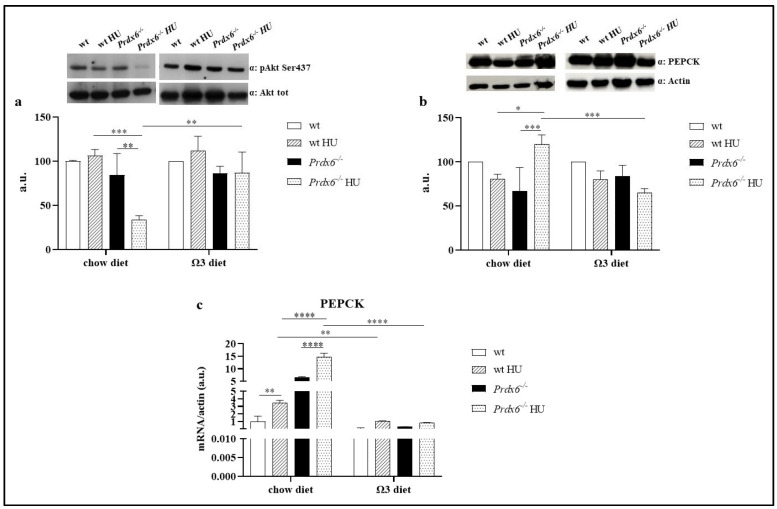
Insulin pathway activation assessment. (**a**) Phospho Akt1 at Ser473 levels. (**b**) PEPCK protein levels. (**c**) Gene expression related to PEPCK. Data are reported as mean ± SD. Graphs represent one of three separate studies, all yielding similar results (*n* = 5). * *p* < 0.05; ** *p* < 0.005, *** *p* < 0.001, **** *p* < 0.0001. a.u. = arbitrary unit.

Based on the previous improvement in glucose homeostasis, we move forward by analyzing the insulin signaling activation and gluconeogenesis in the liver of our mouse model. Specifically, we assessed the insulin signaling pathway in both wild-type (wt) and *Prdx6*^−/−^ mice by examining the phosphorylation levels of Akt1 at Ser473, which corresponds to the activated form of Akt1 [18]. As shown in Figure 3a, neither HU (in the chow diet) nor the omega-3-enriched diet had any impact on insulin signaling in wt mice. However, in *Prdx6*^−/−^ HU mice, a significant reduction in Ak1t activation compared to both *Prdx6*^−/−^ mice (*p* < 0.005) and wt HU mice (*p* < 0.001) has been observed, suggesting an impaired insulin activation in this metabolically altered mouse model. Interestingly, the omega-3-enriched diet significantly restored pAkt1 levels in *Prdx6*^−/−^ HU mice compared to *Prdx6*^−/−^ HU mice fed with a standard diet (*p* < 0.005). Taken together, these data suggest that omega-3 supplementation may be beneficial in counteracting alterations related to insulin signaling. To further validate the reported effect of omega-3 on insulin pathway, we also analyzed the expression levels of a rate-controlling enzyme on gluconeogenesis, PEPCK. It has been demonstrated that increased levels of PEPCK, by enhancing gluconeogenesis, promote hepatic glucose release, contributing to hyperglycemia in both human and animal models [19]. According to the observed reduced pAkt1 levels in *Prdx6*^−/−-^ HU mice, we reported a significant increase in PEPCK levels in these mice when fed a chow diet, compared to both *Prdx6*^−/−^ mice (*p* < 0.001) and wt HU mice (*p* < 0.05) (Figure 3b). Nevertheless, when an omega-3-enriched diet was administered before HU, *Prdx6*^−/−^ HU mice displayed significantly reduced PEPCK levels compared to *Prdx6*^−/−^ HU mice fed with a standard diet (*p* < 0.001). This finding was also confirmed by evaluating the gene expression levels of PEPCK in the liver of our mouse model. As shown in Figure 3c, HU significantly increased PEPCK expression in both wt and *Prdx6*^−/−^ mice compared to their control group (*p* < 0.005 and *p* < 0.0001, respectively). Furthermore, the administration of an omega-3-enriched diet resulted in a reduction in PEPCK expression in the HU condition compared to their counterparts on a standard chow diet (*p* < 0.005 and *p* < 0.0001, respectively), confirming the beneficial impact of omega-3 against the effects of microgravity.

## 4. Discussion

With the advancement of technological innovation in the field of aerospace engineering, the once-remote possibility of traveling, living, and working in space is becoming more real. However, microgravity induces some alterations that may result in pathological conditions, which should be considered when planning space trips. A recent review has comprehensively described the impact of space flight on human health, including the effects of radiation that increases oxidative stress, isolation, which can influence mental health leading to depression and anxiety, as well as microgravity that contributes to body fluid redistribution and metabolic alterations [20]. Among these alterations, it is well established that both space flight and the bed rest experimental procedures induce glucose intolerance and insulin resistance [3,21]. Similarly, Gambara and coworkers demonstrated a dysregulation in several genes involved in insulin sensitivity in mice exposed to microgravity [22]. According to these findings, our preliminary results demonstrated that *Prdx6*^−/−^ mice subjected to hindlimb unloading (HU) displayed disrupted glucose homeostasis, characterized by an increase in glucose and insulin secretion levels, suggesting the presence of insulin resistance.

Insulin decreases glucose release by inhibiting PEPCK, a key regulatory enzyme of gluconeogenesis, through the activation of the phosphatidylinositol 3 (PI 3-kinase)/Akt-dependent pathway [23]. Therefore, when insulin resistance occurs, as in microgravity, this inhibition is diminished, resulting in an increase in PEPCK. In agreement, *Prdx6*^−/−^ mice subjected to HU exhibited impaired insulin signaling, characterized by a significant decrease in Akt activation, which consequently led to an increase in PEPCK protein and gene expression. These findings were consistent with the results of Vitry and coworkers [24] who reported a decrease in Akt-mediated pathway in the quadriceps of mice belonging to the NASA Rodent Research 1 (RR1) group, and with Ramirez and colleagues [25], who reported a significant increase in PEPCK expression in the liver of mice subjected to HU.

To counteract the detrimental effects of space flights, several nutritional approaches have been proposed [21]. Specifically, an omega-3-enriched diet has been recommended to mitigate the bone loss observed in microgravity [9]. At the same time, a low-glycemic-index dietary approach has been proposed to ameliorate glucose-related alterations. Although a beneficial role of omega-3 in mitigating insulin resistance has also been reported [8], there are currently no data available regarding its effects on glucose metabolism during spaceflight. In the present study, we reported that an omega-3-enriched diet could partially counteract the alterations in glucose homeostasis induced by microgravity. Indeed, both the glucose and insulin AUC were significantly decreased in *Prdx6*^−/−^ mice exposed to HU. In line with those evidences, an improvement in Akt activation was also observed, in association with a correlated reduction in the protein and gene expression of PEPCK. Furthermore, the ground-based effect of omega-3 in reducing PEPCK levels and gluconeogenesis, resulting in an improvement of glucose homeostasis, has been previously reported [26]. Our preliminary results highlight the relevant role of a nutritional approach based on omega-3 to mitigate the glucose metabolic alteration resulting from exposure to microgravity. Our study’s strength lies in the use of a model of both aging and metabolic alterations closely resembling those observed in individuals, especially old rich men, considering a space flight and exposure to microgravity effects. Our study’s strength is in the use of a model of aging and metabolic disorders that may represent the health status of the future civilian travelers.

However, there are some limitations to the present study that should be addressed. Firstly, using a genetically modified animal model may induce compensatory mechanisms that need to be considered. Additionally, as clearly indicated, these are only preliminary results, and further in-depth analyses are required to better elucidate the mechanisms linking omega-3 and glucose metabolism in a microgravity environment. Moreover, another significant limitation is the absence of data related to oxidative stress. Considering the mouse model utilized and the exposure to microgravity, oxidative stress may play a pivotal role in the effects on glucose metabolism. Further in-depth studies are needed to fully evaluate the involvement of oxidative stress (including reactive oxygen species, endogenous antioxidants, and corticosterone levels) in the observed effects of both microgravity and an omega-3-enriched diet.

## 5. Conclusions

Soon, it will be possible to travel, live and work in space. However, it will be essential to consider nutritional approaches that can mitigate the adverse effects of microgravity, particularly those related to glucose homeostasis. Our preliminary results suggest that an omega-3-enriched diet may be helpful in alleviating the effects of microgravity, especially in people with pre-existing or latent metabolic alterations.

## Data Availability

All data are contained within the manuscript.
